# The Medical Portrait Gallery

**Published:** 1840-04

**Authors:** 


Art. XIV.—*The Medical Portrait Gallery.* By J. S. Pettigrew, f.r.s. Vol. IV.—*London,* 1840.

The last Part (xxv.) of this publication has just made its appearance, and completes, we are sorry to say, not merely the volume but the work. We were in hopes that the encouragement extended to Mr. Pettigrew's labours would have enabled him to proceed for another year or two; as we, in common with many others, were anxious to possess good likenesses of many other friends, ornaments of the profession, who probably would have come within the scope of the author's plan. As we have freely criticised this work on former occasions, with the good intention of improving it, we cannot finally dismiss it without recommending it as an elegant, amusing, and instructive publication. It ought to be in the library of every member of the profession, at least, who either cares for handsome books or loves to encourage literary enterprise.

This volume contains portraits and memoirs of Galen, Harvey, Cullen, Brown; of Sir James M'Grigor, Drs. Hall, Powell, Roget, Young; and Messrs. Copeland, Guthrie, Pettigrew, and Stafford. The whole of the last Part is occupied by an autobiography of the author, which is extremely amusing.

